# Beyond Spherical: Unveiling the Significance of Oval Blastocyst Morphology on Euploidy and Implantation Success

**DOI:** 10.3390/cells14181468

**Published:** 2025-09-19

**Authors:** Jakub Wyroba, Agnieszka Kuczyńska, Klaudia Kasperkowicz, Katarzyna Kostarczyk, Pawel Kordowitzki, Joanna Kochan

**Affiliations:** 1 Malopolski Institute of Fertility Diagnostics and Treatment—KrakOvi, 30-118 Krakow, Poland; jakub.wyroba@krakovi.med.pl (J.W.); klaudia.kasperkowicz@krakovi.med.pl (K.K.); 2 Fertility Disorders Clinic, Andrzej Frycz Modrzewski Krakow University, 30-705 Krakow, Poland; 3 Kriobank Infertility Treatment Center, 15-879 Bialystok, Poland; agnieszka.kuczynska@kriobank.pl; 4 Department of Animal Reproduction, Anatomy and Genomics, University of Agriculture in Krakow, 31-120 Krakow, Poland; katarzyna.kostarczyk@student.urk.edu.pl; 5 Institute of Veterinary Medicine, Nicolaus Copernicus University, 87-100 Torun, Poland; p.kordowitzki@umk.pl; 6 Department of Gynecology, Charité Medical University, 10117 Berlin, Germany

**Keywords:** oval blastocysts, blastocyst shape, embryo morphology, PGT-A, ICSI

## Abstract

The selection of the most suitable embryo, based on the morphology and shape, for embryo transfer is a critical aspect of the in vitro fertilization (IVF) process, as its precision can significantly enhance the overall effectiveness of IVF and contribute to a healthy birth. This study aimed to compare the chromosomal status and implantation potential of oval-shaped blastocysts versus normal-shaped blastocysts on day 5 post-ICSI (intracytoplasmic sperm injection). Initially, the frequency of oval blastocysts was assessed by analyzing 1328 embryos from 610 ICSI cycles. Subsequently, 80 patients undergoing ICSI and PGT-A (preimplantation genetic testing for aneuploidy), who had both normal and oval blastocysts in the same cycle, were selected to evaluate the euploid rate relative to blastocyst morphology. Finally, the implantation outcomes of fresh embryo transfers involving oval and normal-shaped blastocysts, neither of which had undergone PGT-A, were analyzed. Half of the blastocysts from each group were transferred after assisted hatching (AH), and the other half were transferred without AH. Blastocyst shape does not appear to correlate with an increased risk of aneuploidy but does influence hatching ability. Following AH, the implantation potential of elongated blastocysts is equivalent to that of normally shaped blastocysts, suggesting AH is beneficial for oval embryos. Consequently, the transfer of oval blastocysts is considered as safe and effective as the transfer of normally shaped embryos.

## 1. Introduction

**Figure 1:**
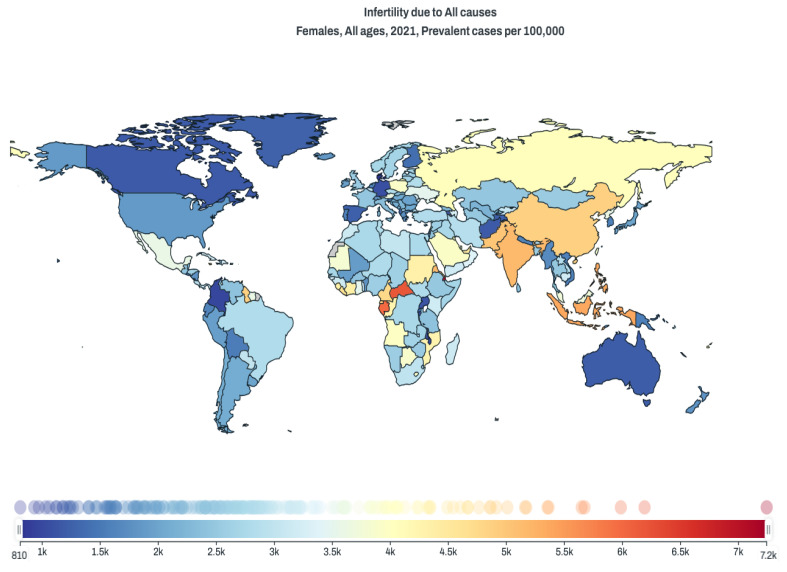
Picture showing the global infertility prevalence in 2021, according to the Database of the Institute for Health Metrics and Evaluation. Prevalence is shown in thousands “k” on the *x*-axis per 100,000 inhabitants.

Female infertility is still a global clinical issue with moderate prevalence (Figure 1), and one of the most important decisions made during the in vitro fertilization (IVF) procedure is the selection of a proper embryo for embryo transfer (ET), as the accuracy of this decision may increase the overall efficiency of the entire IVF procedure and the birth of a healthy child. For many years, the basic tool for this selection has been the morphological assessment based on the Gardner scale, which considers the degree of expansion of the blastocyst, as well as the morphology of its inner cell mass (ICM) and trophectoderm (TE) [1]. More recently, this morphological assessment has been supported by the analysis of embryo morphokinetics using a time-lapse system [2]. Preimplantation genetic testing for aneuploidy (PGT-A) is a powerful tool for selecting euploid embryos and has been used for over a decade [3]. Despite its undeniable advantages, however, PGT-A is an invasive and expensive test that is not authorized in all countries. Therefore, despite these technological developments, some embryos are still transferred solely on the basis of their morphology. This includes a large number of cryopreserved embryos that were stored before these other developments were available. Primarily, a large number of untested embryos are transferred that were cryopreserved many years ago and assessed only on the basis of the Gardner scale [4]. Therefore, non-invasive markers predicting the health of the embryo and its implantation potential are still being sought. For example, it has been shown that the degree of blastocyst expansion is positively correlated with euploid rate [5] and implantation potential [6,7]. Beyond the degree of expansion, other morphological and morphometric parameters of the blastocyst that have been analyzed include inner cell mass size and shape, and the ratio of the size of the ICM (inner cell mass) to the blastocyst diameter [8,9,10]. Morphological defects during embryo development have also been taken into account, such as cellular fragmentation, blastomere multinucleation, asymmetry of blastomeres, irregular shape, and vacuoles [11,12,13].

In embryological practice, we often find oval-shaped blastocysts. This shape could have arisen as the result of the fertilization of an oval oocyte, or may have appeared during embryo development. Despite the numerous studies on the influence of embryo morphology on the implantation potential and chromosomal status of embryos, there has not been a detailed evaluation of the influence of blastocyst shape. However, there are several conflicting reports regarding the influence of oocyte shape on the results of in vitro fertilization. While Yakin et al. (2007) found no association between an oval oocyte shape and the developmental rate and aneuploidy of embryos [14], another report suggested that oocyte dysmorphism, such as an oval shape, may affect fertilization rate and early embryo development, but not blastocyst quality [15]. The aim of this study was to compare the chromosomal status and implantation potential of oval-shaped blastocysts with normal-shaped blastocysts at day 5 after ICSI (intracytoplasmic sperm injection).

## 2. Materials and Methods

### 2.1. Study Design

The retrospective study was carried out in the Krakovi Clinic in Kraków (Poland) and involved procedures of intracytoplasmic sperm injection (ICSI) from 2021 to 2024 in accordance with the guidelines of the local bioethics committee (KBKA/7-8/O/2024).

The research was conducted in three phases:

**Incidence Determination (phase I):** The initial phase quantified the prevalence of oval-shaped blastocysts by analyzing 1328 embryos across 610 ICSI cycles.

**Euploidy Assessment (phase II):** The second phase involved a cohort of 80 patients undergoing ICSI and preimplantation genetic testing for aneuploidy, where both normal- and oval-shaped blastocysts were present within the same cycle. This allowed for a direct comparison of euploidy rates relative to blastocyst morphology.

**Figure 2:**
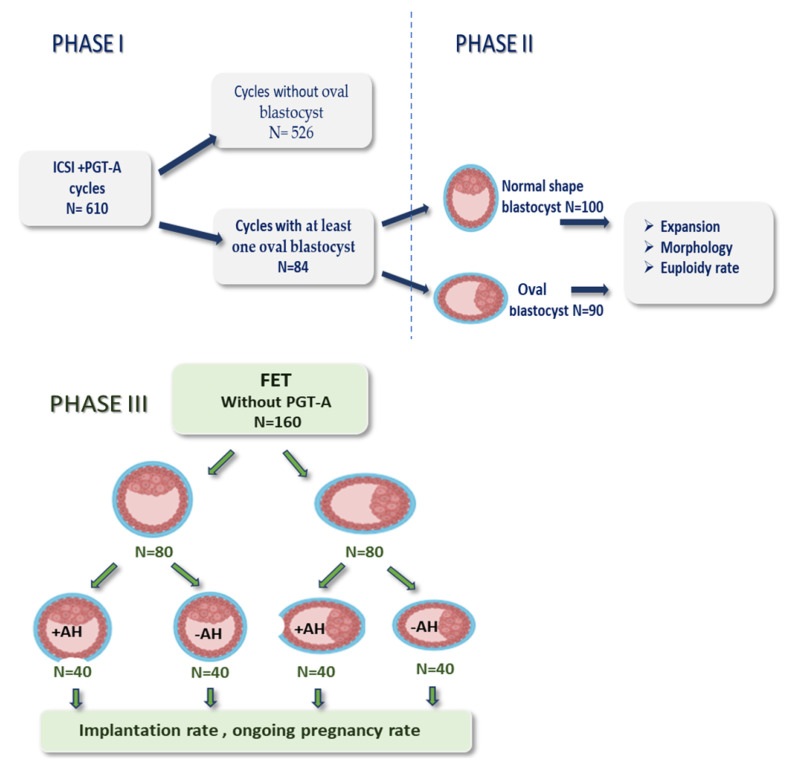
Scheme showing the study design. (Abbreviations: PGT-A-preimplantation genetic testing for aneuploidy, FET-frozen embryo transfer, AH-assisted hatching).

**Implantation Potential Evaluation (phase III):** In this phase, the outcomes of frozen-embryo transfers (FET) involving 80 oval blastocysts and 80 normal-shaped blastocysts were analyzed, none of which had undergone PGT-A, generated from women aged ≤ 38 years to eliminate the influence of advanced maternal age. Within this phase, half of the blastocysts from each morphological group received assisted hatching prior to transfer, while the other half did not. The study design is also depicted in Figure 2.

### 2.2. Clinical Protocols

Patients underwent either a long agonist protocol or a short antagonist protocol, with the choice influenced by their anti-müllerian hormone (AMH), levels and risk of ovarian hyperstimulation syndrome (OHSS).

Long Agonist Protocol:

Patients began treatment approximately one week before their expected menstrual period (cycle days 18–23) with the GnRH (gonadotropin-releasing hormone) agonist, [triptorelin], administered subcutaneously at 1 mg/d. Once pituitary downregulation was confirmed by serum estradiol (E2) levels below 40 pg/mL, ovarian stimulation commenced. This involved a daily subcutaneous dose of [150–300 IU recombinant follitropin alfa (rFSH)] and, in some cases, an additional [75–150 IU menotropin (hMG—human menopausal gonadotropin)].

Antagonist Protocol:

In this protocol, a GnRH antagonist, either [Cetrorelix (Cetrotide Merck Europe, 0.25 mg/d)] or [Ganirelix Gedeon Richter 0.25 mg/d], was administered subcutaneously. Its initiation coincided with the largest follicle reaching a diameter of 14 mm. [rFSH/hMG] administration began earlier, on day 2–4 of the cycle.

Both agonist and antagonist protocols continued until the day of human chorionic gonadotropin (hCG) administration. This trigger occurred when the leading follicle reached at least 18 mm in diameter, and at least three follicles measured 17 mm or more. At this point, [rFSH] was stopped, and a single subcutaneous bolus of either [10,000 IU hCG] or [6500 IU rhCG] was given 36 h prior to scheduled oocyte retrieval. In antagonist cycles with a risk of OHSS, a 2 mg subcutaneous bolus of [triptorelin] was used as a trigger, followed by a freeze-all policy. All follicles measuring 12 mm or larger were aspirated.

Ovarian Stimulation Monitoring in ICSI:

Monitoring included baseline blood sampling and transvaginal sonography on cycle day 2 or 3. Ovarian response was subsequently monitored with TVS (transvaginal ultrasound) and hormonal analysis (E2, FSH, LH) on cycle days 2–3; E2 on days 5–6 and 8–9; and E2, P4 on the day of hCG administration. Additional TVS monitoring was performed as clinically indicated.

Frozen Embryo Transfer (FET):

Endometrial priming involved oral E2, starting on day 1, 2, or 3 of the cycle, with an incremental dosage (2 mg/day for days 1–7, 4 mg/day for days 8–12, 6 mg/day from day 13 until transfer). After 12–14 days of E2, a vaginal ultrasound confirmed endometrial thickness (>7 mm) and absence of a leading follicle. Progesterone (P4) supplementation was then initiated, and FET was scheduled. For natural cycles (t-NC), TVS on day 2 or 3 ruled out cysts or corpus luteum. Cycles were canceled if serum P4 exceeded 1.5 ng/mL on day 2 or 3. TVS monitoring in t-NC began around day 8–10, with endocrine monitoring (E2, LH, P4) once the leading follicle reached approximately 15 mm. Frequent monitoring helped precisely time ovulation for FET scheduling. Clinical pregnancy was confirmed by an intrauterine gestational sac with fetal cardiac activity at 8 weeks, while ongoing pregnancy was defined as completion of over 12 weeks of gestation with fetal cardiac activity.

### 2.3. Laboratory Protocols

Oocyte–cumulus complexes were identified, washed, and incubated for approximately 3 h in washing medium (Wash, Gynemed, Sierksdorf, Germany) under specific atmospheric conditions (6.0% CO_2_, 5.0% O_2_). Cumulus cells were then removed using hyaluronidase and mechanical pipetting. Only metaphase II oocytes with a first polar body were selected for further procedures. Intracytoplasmic sperm injection was performed using an RI Integra 3 micromanipulator following standard techniques.

Embryos were cultured for 5 days in SAGE^®^ medium (Origio, Mumbai, India) at 37 °C under a 6.0% CO_2_, 5.0% O_2_, and balanced nitrogen atmosphere. Embryo development was assessed daily, and blastocysts were graded using the Gardner scoring criteria. Blastocyst biopsy involved the same micromanipulator and microscope as ICSI, with zona pellucida perforation performed using an Octax^®^ laser for 250 μsec. Biopsied trophectoderm cells were washed with D-PBS and placed in 0.2 mL PCR tubes for next-generation sequencing by Igenomix Inc. (Miami, FL, USA). Blastocysts were vitrified using Kitazato^®^ (Shizuoka, Japan) media and the Cryotop device according to the manufacturer’s instructions. For transfer, blastocysts were warmed in Kitazato media for at least 1.5 h, then placed in either EmbryoGlue^®^ medium, (Vitrolife, Gothenburg, Sweden) or SAGE^®^ medium, with over 95% of transfers utilizing Embryo Glue^®^ medium. Half of the blastocysts from each group were transferred after assisted hatching (AH), and the other half without.

All laboratory procedures were performed by experienced, certified embryologists using the same equipment and were performed according to the same protocols.

### 2.4. Statistical Analysis

Non-parametric data, specifically differences in percentage values between groups, were analyzed using the chi-squared test. Parametric data were expressed as means ± SD and compared by two-way ANOVA. Differences were considered significant when the *p*-value was ≤0.05. The statistical analysis was performed using PQStat 1.6.2 (PQStat Soft, Poznan, Poland).

## 3. Results

**Figure 3:**
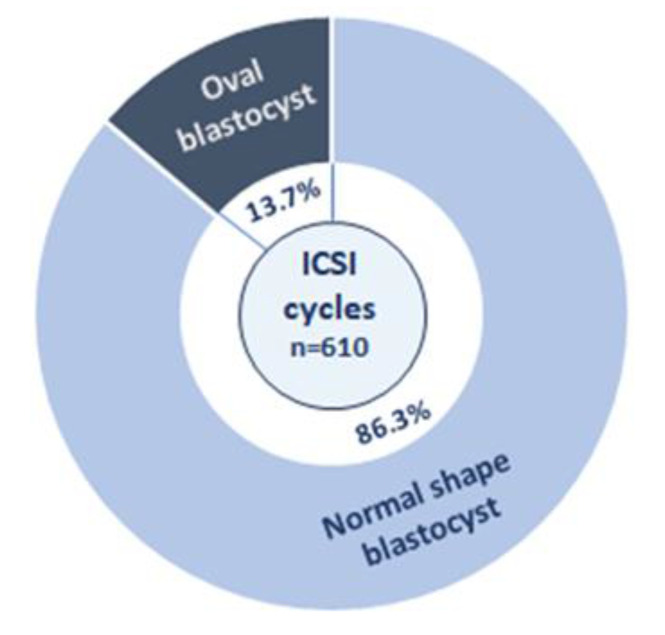
Graph showing the rate of normal-shaped (light blue) and oval-shaped blastocysts (dark blue) in 610 ICSI cycles.

**Figure 4:**
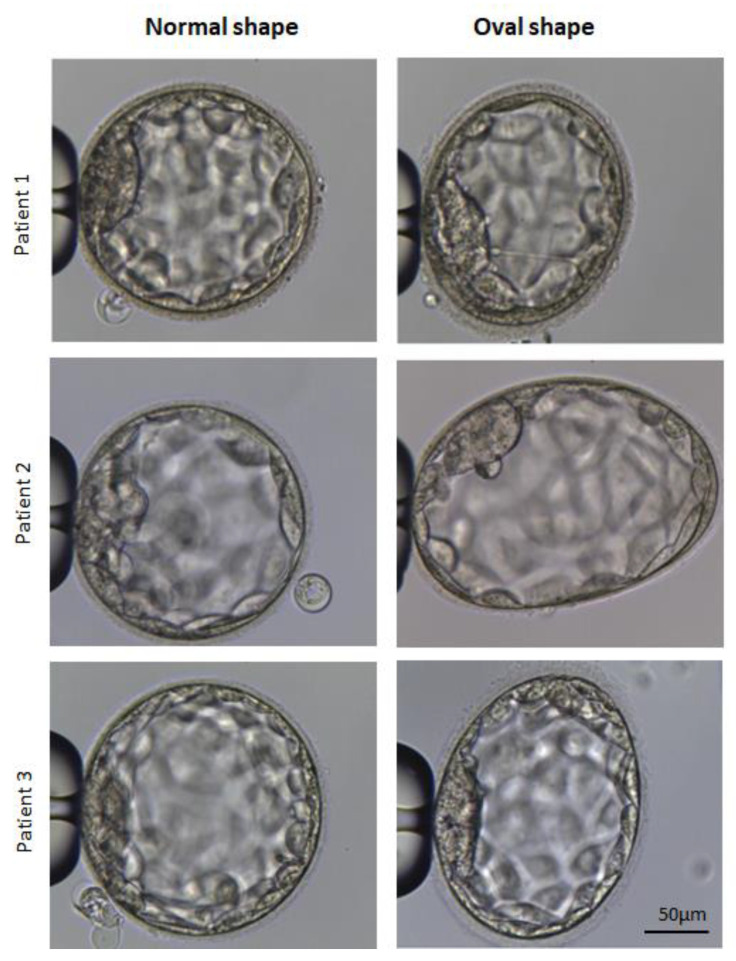
Representative examples of blastocysts with normal and oval shapes obtained from the same cycle within each patient.

The initial phase of the investigation involved analyzing 1328 blastocysts from 610 intracytoplasmic sperm injection cycles to ascertain the prevalence of oval blastocysts (Figure 3). The incidence of oval blastocysts (Figure 4), relative to all blastocysts, was found to be 7.3%, with at least one oval blastocyst observed in 13.7% of the cycles. Baseline characteristics of the cycles examined in this stage are shown in Table 1. No significant correlations were identified between maternal age, body mass index, or ovarian reserve and the occurrence of oval embryos.

In the subsequent phase, 190 blastocysts that had undergone preimplantation genetic testing for aneuploidy from 80 patients were selected to compare the euploid rates of elongated versus normally shaped blastocysts. Each patient provided at least one oval and one normally shaped embryo for analysis, with an average of 2.3 ± 0.2 blastocysts per patient. While no significant morphological differences were noted between the groups beyond the elongated shape (Table 2). Furthermore, no differences in the euploid blastocyst rate were detected between the embryos from the two groups.

**Figure 5:**
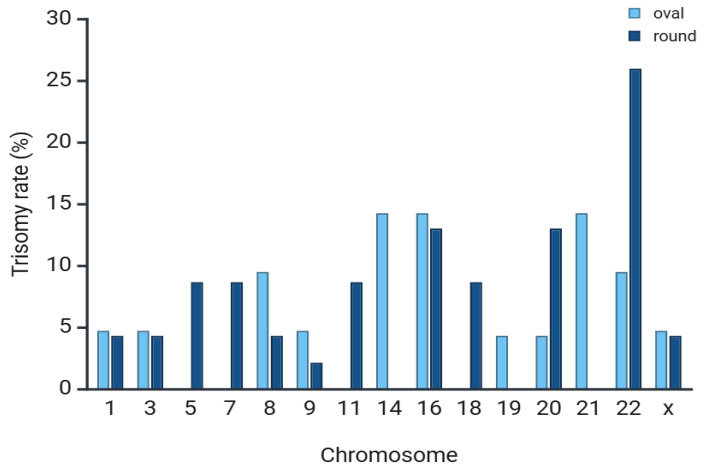
Graph showing the trisomy rate in % of single chromosomes in the respective blastocyst groups. In light blue, the trisomy rate of oval blastocysts is shown, and in dark blue, the trisomy rate of normal round-shaped blastocysts is shown.

The trisomy rate in the respective blastocyst groups differed with regard to the affected chromosomes, as shown in Figure 5.

Finally, a comparison of frozen embryo transfer outcomes between normally shaped and oval-shaped embryos was conducted (Table 3). A significant difference in implantation potential was found after FET of oval and normal-shaped embryos when assisted hatching was not performed. However, following AH, no differences in implantation rates were observed based on blastocyst shape. Additionally, no differences in ongoing pregnancy rates were found between the groups. These findings collectively suggest that blastocyst morphology, specifically an oval shape, does not inherently indicate aneuploidy but rather impacts the blastocyst’s ability to hatch, highlighting the potential utility of assisted hatching to optimize implantation outcomes for such embryos.

## 4. Discussion

The aim of our study was to answer the question of whether oval blastocysts are equally suitable for selection for ET as those with a regular shape. In embryological practice, although we often observe oval blastocysts, they are not usually preferentially selected for ET due to the lack of information regarding the likely outcome, and they are transferred only if the patient does not have any normally shaped embryos. The elongated shape of blastocysts in most cases is a consequence of the fertilization of oval oocytes. The structure responsible for maintaining the round shape of the oocyte and the subsequent embryo is the zona pellucida (ZP), and its spherical shape ensures maximum contact between the blastomeres of the embryo [16].

Two hypotheses can be assumed for the formation of oval oocytes. The first assumes that oocytes are mechanically elongated as the result of improperly performed oocyte retrieval or denudation procedures. However, it appears that most oocytes undergo elongation in the follicle, e.g., during oocyte development and secretion of the zona proteins [16]. We expected that oocyte elongation would also be related to oocyte aging, ZP dysfunction, and loss of oocyte turgor with age, as various oocyte anomalies have been reported to appear with age [17]. While we had assumed that older patients would have a higher incidence of oval embryos due to oocyte aging and changes in turgor and ZP structure, we found that the frequency of oval blastocysts was not age dependent. To date, there are no reports on the frequency of oval blastocysts in IVF/IVC procedures, and there are very different reports regarding the occurrence of oval oocytes (5.9–17.9%) [15]. These discrepancies depend on whether we count the percentage of cycles with at least one oval oocyte or the ratio of oval oocytes to all oocytes obtained.

Here, we found that 13.7% of cycles had at least one oval blastocyst, and that the incidence of oval blastocysts as a proportion of all blastocysts was 7.3%. Ebner et al. reported 17.9% of cycles with at least one ovoid MII oocyte, which is slightly higher than our results [15]. The lower percentage of oval blastocysts than oval oocytes is logical, though, because not all oocytes, regardless of their shape, will develop to the blastocyst stage. Most of the oval blastocysts analyzed in this study developed as a consequence of fertilization of oval oocytes. We observed an individual predisposition towards the occurrence of oval oocytes and then blastocysts, regardless of the patient’s age, and patients who had oval blastocysts in one cycle also had them in each subsequent cycle. Moreover, we observed that patients often had several oval blastocysts in one cycle (unpublished data). In this report, we only analyzed one cycle for each patient

The correlation between an oocyte’s shape and its likelihood of fertilization and subsequent embryo development is still unclear. There are conflicting reports in the literature regarding the developmental potential of oval oocytes, with some authors suggesting that oocyte dysmorphism, such as oval shape, may affect fertilization rate and embryo development [15,18,19,20]. while others found no significant difference in these outcomes between normal-shaped and oval-shaped oocytes [21,22]. According to Ebner et al. 2008, elongated oocytes with an abnormal cleavage pattern show delayed preimplantation development and the degree of dysmorphism was found to be related to cleavage pattern [15]. In contrast, Dolgushina et al. 2015 reported that the morphokinetic patterns of ovoid oocytes were similar to those of normally shaped oocytes [23].

However, there are no direct reports on the morphology, euploidy and implantation potential of oval blastocysts. In our investigation, we did not observe any correlation between the morphological quality of the blastocysts and their shape. However, there were significantly more hatching blastocysts among the normal-shaped blastocysts compared to the oval group (31% vs. 12%, *p* < 0.001). This may confirm theories about the significant role of ZP during the hatching process [16]. The zona pellucida serves a critical multifaceted role throughout early mammalian embryonic development. Initially, it is integral to the fertilization process by preventing polyspermy. Subsequently, it acts as a protective encapsulation for the developing embryo, maintaining homeostatic conditions with its external milieu. In the preimplantation phase, the ZP undergoes a process of dissolution, facilitating embryonic hatching and subsequent uterine implantation. During blastocyst expansion, the accumulation of fluid within the blastocoel, driven by the activity of the Na^+^/K^+^ pump in the outer layer of trophectoderm cells (TEs) pump in the trophectoderm cells, exerts mechanical pressure on the ZP. This pressure, combined with the enzymatic action of proteases secreted by TE cells, leads to the softening, thinning, and ultimate rupture of the ZP [24,25,26]. Therefore, the dysfunction of the ZP that causes oocyte elongation may also be related to the altered hatching process of the blastocyst developing from this oocyte.

The results of our study indicate that abnormal shape has no correlation with embryo euploidy. This is consistent with a previous report, in which the FISH method was used [14]. Also, similar types of chromosomal aberrations (trisomy, monosomes) were found in both normally and abnormally shaped aneuploid embryos. After FET of blastocysts without PGT-A testing or assisted hatching, elongated embryos had a lower implantation potential compared to those with a round shape (50% vs. 63%). This may confirm the observations from the earlier stage of our study, where we observed a significantly higher rate of spontaneous hatching in embryos with a normal shape than in those with an oval shape, and confirm that the zona structure might affect the hatching and/or implantation process.

A limitation of our study is that the analysis of implantation potential included the transfer of blastocysts that had not undergone PGT-A. Although it would have comprised a more homogeneous group if we had been able to include only euploid oval embryos in this assessment, this group was too small to allow for a reliable statistical analysis. The advantage of our research is that it was conducted in one center, following exactly the same protocols, which ensured the repeatability of the procedures. The second major advantage of our study is that the same patients served as study and control groups because at least one oval and one normal-shaped embryo was analyzed from each patient.

## 5. Conclusions

In conclusion, blastocyst shape is not associated with a higher risk of aneuploidy but is related to the hatching ability of the blastocyst, which can make the implantation process more difficult. After assisted hatching, the implantation potential of elongated blastocysts is the same as that of blastocysts with a normal shape, so AH seems justified for oval embryos. Therefore, transfer of oval blastocysts is as safe and effective as that of embryos with a normal shape.

## Figures and Tables

**Table 1:** **Baseline characteristics of patients/cycles included in the analysis of the frequency of occurrence of oval blastocysts.** * Normal shape blastocyst—cycles without oval blastocysts, ** Oval shape blastocysts- cycles with at least one oval blastocyst, AMH—Anti-Mullerian hormone, BMI—the body-mass index is the weight in kilograms divided by the square of the height in meters.

Parameters		Normal Shape Blastocysts *	Oval Shape Blastocysts **	Total
No of patients (cycle), n (%)		526 (86.3%)	84 (13.7%)	610 (100%)
Age (years), range, mean ± SD		34.9 ± 4.7	35.2 ± 4.4	35.1 ± 4.5
BMI (kg/m^2^), range, mean ± SD		22.4 ± 2.4	22.1 ± 2.5	22.3 ± 2.4
AMH (ng/mL), range, mean ± SD		3.3 ± 1.98	3.1 ± 2.26	3.17 ± 2.12
No of blastocysts, n (%)		1233 (92.7%)	95 (7.3%)	1328 (100%)
No of blastocysts/cycle, mean ± SD		2.3 ± 0.3	1.13 ± 0.2	2.17 ± 0.3
Cause of infertility	Female factor, n (%)	267 (51%)	41 (49%)	308 (50%)
	Male factor, n (%)	47 (9%)	9 (11%)	56 (10%)
	Combined, n (%)	212 (40%)	34 (40%)	246 (40%)

**Table 2:** Morphology and euploidy rate depending on the shape of the blastocyst. Excellent quality (Bl 4AA, Bl 5AA), good quality (Bl 4AB, 4BA, 5AB, 5BA, medium quality (Bl 4BB, Bl 5BB). ^a,c^, values with different superscripts within the same rows differ significantly (*p* < 0.001).

Parameters	Blastocysts	
	Oval Shape	Normal Shape
No of blastocysts, n	90	100
No of expanding (BL 4) blastocysts, n (%)	79/90 (88%) ^a^	69/100 (69%) ^c^
No of hatching (BL 5) blastocysts, n (%)	11/90 (12%) ^a^	31/100 (31%) ^c^
No of excellent quality, n (%)	33/90 (37%) ^a^	41/100 (41%) ^a^
No of good quality, n (%)	34/90 (38%) ^a^	33/100 (33%) ^a^
No of medium quality, n (%)	23/73 (25%) ^a^	26/100 (26%) ^a^
Euploid rate, n (%)	46/90 (51%) ^a^	53/100 (53%) ^a^
Trisomy, n (%)	26/46 (56%) ^a^	28/53 (53%) ^a^
Monosomy, n (%)	20/46 (44%) ^a^	25/53 (47%) ^a^

**Table 3:** **Comparison of implantation and ongoing pregnancy rate based on blastocyst shape.** AH- assisted hatching; ^a,b^-values with different superscripts within the same rows differ significantly (*p* < 0.05).

Parameters	FET	
	Normal Shape	Oval Shape
No of FET	80	80
Patient age (years), mean ± SD	32.1 ± 2.3	32.3 ± 2.6
Patient BMI (kg/m^2^), mean ± SD	21.9 ± 2.2	22.1 ± 2.1
Implantation rate without AH, n (%)	25/40 (63%) ^a^	20/40 (50%) ^b^
Implantation rate with AH, n (%)	24/40 (61%) ^a^	25/40 (62%) ^a^
Total implantation rate	49/80 (62%) ^a^	45/80 (56%) ^a^
Ongoing pregnancy rate, n (%)	46/80 (57%) ^a^	42/80 (52%) ^a^

## Data Availability

Data can be requested via email to the corresponding author.
